# Health-Related Quality of Life in Ankle Osteoarthritis: A Case-Control Study

**DOI:** 10.1177/19476035211025814

**Published:** 2021-06-24

**Authors:** Liam D.A. Paget, Johannes L. Tol, Gino M.M.J. Kerkhoffs, Gustaaf Reurink

**Affiliations:** 1 Amsterdam UMC, University of Amsterdam, Department of Orthopaedic Surgery, Amsterdam Movement Sciences, Amsterdam, The Netherlands; 2 Academic Center for Evidence-Based Sports Medicine (ACES), Amsterdam, The Netherlands; 3 Amsterdam Collaboration for Health and Safety in Sports (ACHSS), AMC/VUMC IOC Research Center, Amsterdam, The Netherlands; 4 Aspetar, Orthopaedic and Sports Medicine Hospital, Doha, Qatar; 5 The Sport Physician Group, Department of Sports Medicine, OLVG, Amsterdam, The Netherlands

**Keywords:** autologous blood, grafts, intra-articular delivery, ankle, osteoarthritis

## Abstract

**Objective:**

Ankle osteoarthritis (OA) has a prevalence of 3.4% in the general population of which 70% to 78% is posttraumatic, affecting younger patients with a longer projected life span compared with hip and knee OA. The current literature reports the physical and mental quality of life (QoL) of patients with ankle OA, to be similar to end-stage hip OA, end-stage kidney disease and digestive heart failure. However, the QoL of ankle OA patients has not yet been determined compared with a matched control group representing the general population. Our aim is to determine the physical and mental QoL compared with a matched control group.

**Design:**

The Physical and Mental Component Summaries of the Short Form–36 of 100 patients with ankle OA were compared with 91 age- and gender-matched controls. This case-control study is a substudy of the PRIMA trial, in which the efficacy of platelet-rich plasma injections for ankle OA is determined.

**Results:**

A clinically relevant difference was found for both the Physical (*P*=0.003; 95% CI −6.7 to −1.3) and Mental Component Summary scores (*P* < 0.001; 95% CI −10 to −6). Patients with ankle OA had a median of 45 points (matched controls 52 points) and 43 points (matched controls 53 points) for the Physical and Mental Component summary scores, respectively.

**Conclusions:**

Patients with ankle OA had a clinically relevant poorer mental and physical QoL compared with matched controls from the general population. Furthermore, the physical QoL of patients with ankle OA from younger age categories was affected more than those in older age categories.

## Introduction

Osteoarthritis (OA) is the most common joint disease and is characterized by pain and disability.^1,2^ The pathogenesis of OA involves mechanical, inflammatory, and metabolic factors and an imbalance between destruction and repair of the joint.^3,4^ Clinically and radiographically determined ankle OA has a prevalence of 3.4% in the general population.^5^ In former professional football and rugby players, the prevalence of ankle OA is 9% to 19% and 4.6%, respectively.^6-9^ The majority of ankle OA cases are posttraumatic (70%-78%) and affect younger patients with a longer projected life span unlike hip and knee OA.^10,11^ Other secondary causes (including rheumatoid arthritis [5%-12%], hemochromatosis [0%-3%] hemophiliac [1%-2%], septic [1%-2%], congenital [2%], and osteonecrosis [1%-2%]) and primary OA (7%-9%) are reported to be the underlying causes of the ankle OA cases that are not posttraumatic.^10,11^

Patients with ankle OA have a poor physical and mental quality of life according to 2 studies in North America.^12,13^ The first study followed 195 patients (matched controlled to 95 subjects) with moderate to severe ankle OA at their clinic.^12^ They found a similar quality of life as reported for end-stage kidney disease and digestive heart failure. The second study matched controlled 130 end-stage ankle OA patients with end-stage hip OA patients on the waiting list for a hip replacement.^13^ They found a similar physical quality of life and a poorer mental quality of life compared with end-stage hip OA patients.

Currently, only 1 study in the United States compares the quality of life of ankle OA patients to the general population.^12^ Generalization of quality of life outcomes to other population is limited, as differences in quality of life, measured with the Short Form–36 (SF-36), differs between countries for the same diseases.^14,15^ Our aim is to complement the current literature and determine whether the results found in this study are reproducible and comparable to a Dutch population. We will determine the quality of life of ankle OA patients that are willing to participate in a trial on injection therapy. The quality of life was determined using the SF-36 and was compared with a matched control group from among the Dutch population. Our hypothesis is that patients with ankle OA have a poorer mental and physical quality of life compared with the control group.

## Methods

### Study Design

This is a case-control study of ankle OA patients and matched controls from the general population. It is a substudy of the PRIMA trial, a randomized, double-blind, placebo-controlled, multicenter prospective study, designed to determine the efficacy of platelet-rich plasma injections in the management of ankle OA.^16^ We used baseline SF-36 patient data from the PRIMA trial and matched controls from a database of the general population. The PRIMA trial is approved by the Medical Ethics Review Committee Amsterdam Medical Center, the Netherlands (ABR 2018-042, approved July 23, 2018) and registered in the Netherlands trial register (NTR7261). The study was sponsored by the Marti-Keuning Eckhardt Foundation, a nonprofit patient organization.

### Study Population

#### Cases

Patients with ankle OA in 6 hospitals in the Netherlands (2 university medical centers, 2 teaching hospitals, a general hospital [Flevo Hospital], and a focus clinic [Bergman Clinic] were informed about the study. The SF-36 data of patients with ankle OA, acquired at baseline on participation in the PRIMA trial, was compared with a matched control group.

#### Controls

The data of the control group was obtained from the Netherlands Cancer Institute (NKI-AVL).^17^ In 1996, the Netherlands Organization for Applied Scientific Research (TNO) conducted a nationwide survey in order to generate normative data for a study of patient with congenital heart defects. Questionnaires were sent to a randomly selected population from the telephone registry in the Netherlands. The telephone registry includes a smaller percentage of individuals between the ages of 15 and 25 years and a larger percentage of men. An effort was made to correct this imbalance by requesting (in the introductory letter) household members in the age category of 15 to 25 years, to fill in the questionnaire. In total, 1771 questionnaires were returned, a response rate of 68%. Compared with the Central bureau of Statistics, the national sample has a slightly greater percentage of men (56% vs. 49%). The male population was slightly skewed toward the younger age categories. The age distribution of the female population closely matched the total population. From this representative sample of the general Dutch population, controls were randomly matched with PRIMA participants for age and gender (maximum 5-year age difference). No information regarding comorbidities of the control patients was available and was therefore not considered in the analysis.

### Eligibility Criteria

All participants signed an informed consent form before participating in the study.

Patients were eligible for inclusion if they have a severity of ankle OA pain on a visual analogue scale (VAS 0-100 mm) ≥40 mm during daily activities, X-rays (anteroposterior [AP] and lateral views) indicating ≥grade 2 talocrural OA on the van Dijk classification (joint space narrowing, with or without osteophytes)^18^ and are ≥18 years of age. Patients were excluded if they have received injection therapy for ankle OA in the previous 6 months, do not want to receive one of the 2 therapies, have clinical signs of concomitant OA of one or more other major joints of the lower extremities that negatively affects their daily activity level or have had a previous ankle surgery for OA or osteochondral defects <1 year (not including surgery for an ankle fracture in the past).

### Study Measures

#### Outcome Measures

At baseline, before receiving the intervention of the PRIMA trial, the ankle OA patients completed the SF-36 questionnaire.^16^ The SF-36 is validated in the Dutch language and consists of 8 subscales.^17^ All (sub)scales and summary scores go from 0 to 100, where 0 represents low quality of life or function and 100 represents high quality of life or function. These subscales can be summarized into 2 scores, the Physical Component Score and the Mental Component Score, which represent the physical and mental quality of life, respectively. The 4 SF-36 subscales that fall under the Physical Component Score are physical functioning, role limitation due to physical problems, bodily pain, and general health. The other 4 subscales, role limitation due to emotional problems, social functioning, mental health, and vitality, are summarized into the Mental Component Score. The minimal clinically important difference (MCID) of the SF-36 for both the Physical and Mental Component Scores is reported to be 3 points.^19^

#### Primary Outcome Measure

The primary outcome is the Physical and Mental Component Scores of ankle OA.

### Statistical Analysis

Data analysis was performed using the statistical software IBM SPSS V.24.0 for Windows. Depending on normal or nonnormal distribution, data were expressed as means and standard deviation (SD) or median and interquartile range (IQR), as appropriate. Baseline characteristics, including age and gender, were analyzed between groups using descriptive statistics. Physical and Mental Component Scores were calculated for all ankle OA patients and controls, as well as for the predetermined age categories (18-40, 41-60, and >60 years of age). Intergroup comparisons (ankle OA patients vs. controls) were determined using the paired Wilcoxon signed rank test. We considered *P* < 0.05 to be statistically significant.

## Results

### Participants

In total SF-36 data from 100 patients from the PRIMA study were matched controlled with 91 patients from the Netherlands Cancer Institute (NKI-AVL). Of the orginal 100 matched controlled patients, 9 had missing values and could not be used. Unfortunately, no more matched controlled patients could be found in the database of the the Netherlands Cancer Institute (NKI-AVL). In the ankle-OA group, there were 59 males and 41 females compared with the 56 males and 35 females of the control group. The average age was a median of 56 years (IQR: 44-64; min-max: 24-87) in the ankle OA group and a median of 56 years (IQR: 43-63; min-max: 23-90) in the control group.

### Mental and Physical Quality of Life

**Figure 1.**
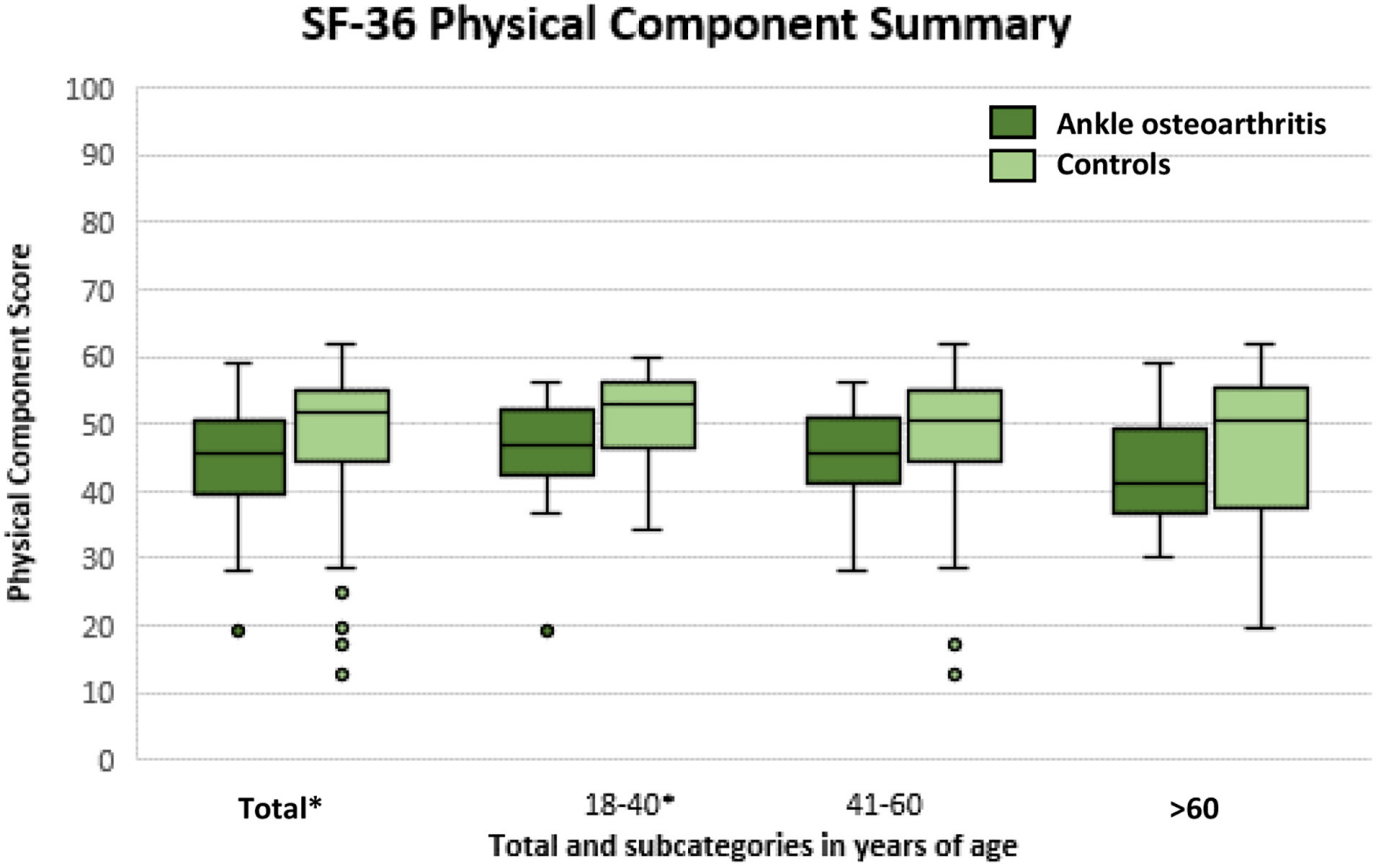
Physical Component Score representing physical quality of life for patients with ankle osteoarthritis (OA) compared with the control group. Statistical significance is illustrated by * on the *x*-axis.

**Figure 2.**
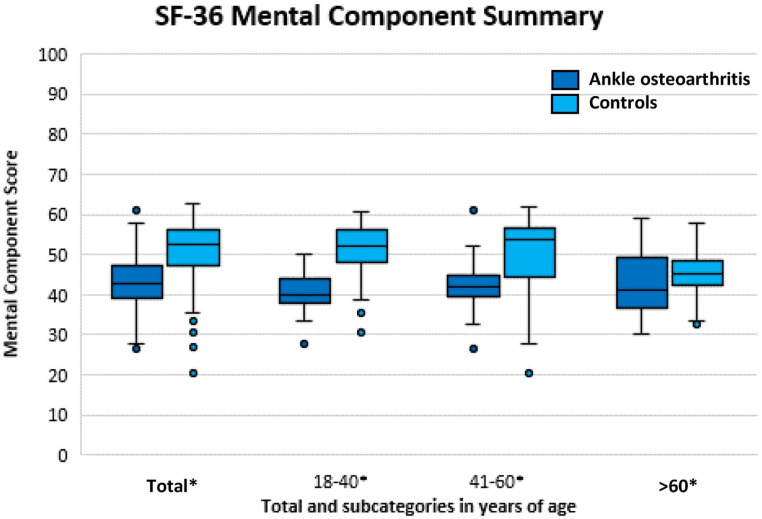
Mental Component Score representing mental quality of life for patients with ankle osteoarthritis (OA) compared with the control group. Statistical significance is illustrated by * on the *x*-axis.

The Mental and Physical Component Scores of the SF-36 for all ankle OA patients and controls, as well as the predetermined age groups (18-40, 41-60, and >60 years) are presented in Figures 1 and 2, respectively (see also Table 1). A statistically significant, and clinically relevant difference was found for the Physical Component Score (*P* = 0.003; 95% CI −6.7 to −1.3), with a median of 45 points (IQR: 40-50; min-max: 19-59) for patients with ankle OA and a median of 52 points (IQR 44-55; min-max: 13-62) for the control group (Figure 1). For the Mental Component Score (Figure 2), a statistically significant and clinically relevant difference was found (*P* < 0.001; 95% CI −10 to−6) with a median of 43 points (IQR: 39-47; min-max: 27-61) for the patients with ankle OA and a median of 53 points (IQR: 47-56; min-max: 20-63) for the control group. For all predefined age categories, a statistically significant and clinically relevant difference was found between ankle OA patients and controls, except for the Physical Component Score of the subgroups 41 to 60 and >60 years of age (Figures 1 and 2).

**Table 1.** Physical and Mental Component Scores for All Ankle OA Patients Compared with Controls and Subdivided into the Predetermined Age Categories.

	Ankle OA, Median (IQR; min-max)	Control, Median (IQR; min-max)	*P*
Total	(*n* = 100)	(*n* = 91)	
Physical Component Score	45 (40-50; 19-59)	52 (44-55; 13-62)	*P* = 0.003 (CI −6.7 to −1.3)
Mental Component Score	43 (39-47; 27-61)	53 (47-56; 20-63)	*P* < 0.001 (CI −10.0 to −6.0)
*Age categories (years)*			
18-40	(*n* = 21)	(*n* = 19)	
Physical Component Score	47 (42-52; 19-56)	53 (46-56; 34-60)	*P* = 0.02 (CI −9.0 to −1.4)
Mental Component Score	40 (38-44; 28-50)	52 (48-56; 31-61)	*P* = 0.005 (CI −14.8 to −5.6)
41-60	(*n* = 47)	(*n* = 43)	
Physical Component Score	45 (41-51; 28-56)	51 (44-55; 13-62)	*P* = 0.10 (CI −7.4 to 0.73)
Mental Component Score	42 (40-45; 27-61)	54 (44-57; 20-62)	*P* < 0.001 (CI −11.4 to −4.97)
>60	(*n* = 32)	(*n* = 29)	
Physical Component Score	41 (37-49; 30-59)	50 (38-55; 20-62)	*P* = 0.16 (CI −8.5 to 1.8)
Mental Component Score	45 (42-48; 32-58)	53 (47-56; 27-63)	*P* = 0.001 (CI −7.1 to −10.4)

OA = osteoarthritis; IQR = interquartile range.

## Discussion

Our most important finding was that ankle OA patients that are willing to participate in a trial on injection therapy have a statistically significant and clinically relevant poorer outcome for both the Physical and Mental Component Scores compared with matched controls from the general population.

We found a poorer Mental Component Score (43 points) compared to the current literature on ankle OA patients (47 points).^12,13^ The Mental Component Score was similar the reported scores in the literature for psychiatric (anxiety disorders, depression, and alcohol abuse/dependence) (44 points)^20^ and cerebrovascular/neurologic conditions (stroke, migraine, multiple sclerosis, neuromuscular disease, Parkinson’s disease, and epilepsy) (44 points).^20^ Compared with knee OA (51)^21^ and hip OA (47),^21^ the Mental Component Score tended to be poorer among ankle OA patients in our cohort. The average age of the patients with knee and hip OA (69 and 67 years) differed considerably from the ankle OA patients in this study (54 years). A potential explanation for the lower Mental Component Scores is that ankle OA patients may be affected more mentally by the physical limitations they experience, as they are generally younger.

For the Physical Component Score (45 points) we found a higher score than the current literature (31 points^12^ and 30 points^13^) on ankle OA. This can be partially explained as being due to the selection of end-stage ankle OA patients in one of the studies, in contrast to our study where we included patients with X-rays indicating ≥ grade 2 talocrural OA on the van Dijk classification.^13^ In the other study, the quality of life of all patients visiting the outpatient clinic was determined.^12^ In our study, ankle OA patients wanted to take part in an injection therapy study (PRIMA trial) as they felt they were not yet ready for surgery (arthrodesis or ankle prosthesis).^18^ Therefore, the difference in reported physical quality of life may possibly be explained by the influence of selection bias. Furthermore, no clinically relevant difference was seen for the Physical Component Scale of the age groups 41 to 60 and >60 years of age. We theorize that it may be due to the accumulation of other physical ailments in patients over the age of 41 years, making the relative impact ankle OA has on the physical quality of life, smaller.

We found similar scores as is reported for knee OA (43) and hip OA (41).^21^ As mentioned earlier, the ankle OA patients in this study were considerably younger (54 years) than patients with knee and hip OA (69 and 67 years). A potential explanation for the higher Physical Component Scores compared with knee and hip OA, is that the relatively younger ankle OA patients are better able to compensate for the physical limitations. The significant difference in age may be explained by the difference in etiology of ankle OA and hip and knee OA.^22,23^ Ankle OA is posttraumatic in 70% to 78% of cases, compared with 2% to 10% in hip and knee OA. Patients with posttraumatic hip and knee OA were also seen to be up to 10 years younger than patients that are not posttraumatic.^24^ Biomechanically, the mortise structure of the ankle joint allows for a higher congruency than the hip and knee joint.^12,22,23^ At cartilage level, that of the ankle is stiffer and less permeable to water, due to a higher content of proteoglycans and lower water content, allowing it to withstand higher loads per unit surface area.^22,25^ The response of ankle cartilage chondrocytes to (catabolic) inflammatory cytokines is also reduced, giving ankle cartilage a higher repair capability, all in all making it possibly less vulnerable to primary OA.^22^

Compared with other diseases in the literature, we found a similar physical component score (45 points) for end-stage chronic kidney disease (44 points).^26^ In this study based on a population in the United Kingdom, the average age was 81 years compared with an age of 54 years in this study.^26^ In further comparison to other diseases, we find a similar Physical Component Score in the literature for cardiovascular (e.g., coronary heart disease and hypertension) (45),^20^ endocrinological (e.g., diabetes and thyroid gland impairment) (44),^20^ and cerebrovascular/neurologic conditions (43).^20^

### Strengths and Limitations

A strength of this study is the large cohort of ankle OA patients and matched controls from the general population. However, there are 2 limitations. First, the ankle OA patients, were subjects who had signed up for the PRIMA trial, and were willing to take part in a blinded randomized controlled trial with 50% chance of a placebo injection. These patients had a minimal ankle OA pain of VAS ≥40 mm during daily activities. It is likely there is a selection bias in that these subjects were impacted more by their ankle OA symptoms than the average ankle OA patient. Second, although considered recent enough, the SF-36 of the matched controls dates back to 1998. In Norway, despite societal changes over a period of 2 decades, the quality of life has remained relatively stable.^27^ We therefore expect this limitation to have negligible consequences.

## Conclusion

Ankle OA patients, that were willing to participate in a trial on injection therapy, had a clinically relevant poorer mental and physical quality of life compared to matched controls from the general population. Furthermore, the physical quality of life of patients with ankle OA from younger age categories was affected more than those in older age categories. Considering the relatively young age, current limited treatment options and especially the considerable impact on the mental quality of life, the outlook of an ankle OA patient is bleak.
